# A path to resilience and social integration: motivations of international student volunteers during the COVID-19 lockdown in Wuhan

**DOI:** 10.3389/fpsyg.2025.1579781

**Published:** 2025-06-03

**Authors:** Meraj Malakouti, Asma Khaleel Abdallah, Ahmed Alkaabi

**Affiliations:** ^1^College of Public Administration, Huazhong University of Science and Technology, Wuhan, China; ^2^Sharjah Education Academy, Sharjah, United Arab Emirates; ^3^College of Education, United Arab Emirates University (UAEU), Abu Dhabi, United Arab Emirates

**Keywords:** volunteering, motivations, resilience, social integration, COVID-19 lockdown

## Abstract

**Introduction:**

The coronavirus disease 2019 (COVID-19) epidemic emerged globally, resulting in the shutdown of schools and universities. The January 2020 lockdown in Wuhan, China, profoundly impacted residents’ daily lives, particularly international students who faced restrictions within their campuses and dormitories. This qualitative study aimed to investigate the motivations of international student volunteers during Wuhan’s COVID-19 lockdown and identify how this voluntary activity fostered resilience and social integration among them.

**Methods:**

The study adopts a qualitative approach using semi-structured in-depth interviews with 14 international PhD students at a university in China who volunteered during the three-month Wuhan lockdown. Data saturation was achieved through purposeful sampling. Data were transcribed, and thematic analysis was applied, with coding using NVivo 14.

**Results:**

After applying thematic analysis, three key themes were identified: (a) motivation for volunteering, (b) resilience through volunteering, and (c) social integration and community bonding through volunteering. These themes were explored in relation to the psychological needs defined in Self-Determination Theory.

**Discussion:**

Our findings further indicate that coping via volunteering not only enhanced psychological resilience but also significantly facilitated social connections among culturally diverse groups. These findings highlight the importance of understanding volunteer motivations and experiences, providing valuable insights for universities and policymakers to develop strategies that enhance student support and community resilience during a crisis.

## 1 Introduction

Natural disasters, including hurricanes, earthquakes, and epidemics, tend to expose the limitations of conventional, vertical aid systems and highlight the need for non-governmental organizations in humanitarian assistance (Hu et al., 2014; Li et al., 2020). Researchers have extensively demonstrated the necessity for coordination among many stakeholders—government, civil society, and citizens—during times of crisis (Kapucu, 2006).

In December 2019, COVID-19 broke out in Wuhan, leading to unprecedented public health measures, most notably a strict lockdown imposed on January 23, 2020, which profoundly interrupted residents’ daily life (Tian et al., 2020). This lockdown, which lasted a total of 76 days, caused widespread fear and panic throughout the city. Residents were not permitted to circulate freely, and Wuhan remained in a state of lockdown until April 8, 2020. While several foreigners were evacuated, international students who chose to remain were confined to their dormitories, unable to leave their rooms or move freely around campus. Students not engaged in volunteer activities, faced strict restrictions, with no opportunities for outdoor activities or campus movement. By contrast, volunteers with certain responsibilities were permitted some movement for community service, so enabling them to interact with others and move around while carrying out their duties. This movement not only allowed volunteers to carry out essential community duties but also gave them these opportunities for outdoor activity, therefore providing physical and mental relief from the strict confinement experienced by non-volunteers. These students, supported by the university, played a vital role in providing essential services and support on campus. These volunteers performed a wide range of tasks, such as food/mask services, handling welfare services, collecting students’ requests, carrying out regular disinfection operations, daily body-temperature checks, providing information about COVID-19 virus as well and providing emotional support. These volunteers had a role as a link between students living on campus and the university authorities.

Despite extensive research on student volunteering, particularly among medical and nursing students, which aimed at providing vital medical assistance and support during crises (Lazarus et al., 2021; Domaradzki and Walkowiak, 2021; Phillips et al., 2022), a significant gap remains in understanding the motivations and contributions of international students during the unprecedented Wuhan lockdown as the first and most unknown case worldwide. As these students found themselves in an unfamiliar and restricted environment, their ability to adapt, contribute, and integrate within the community while facing unprecedented challenges holds paramount importance. Moreover, while Self-Determination Theory (SDT) has been widely used to explore student volunteer motivations (e.g., Haivas et al., 2012; Hamilton Skurak et al., 2019), few studies have applied it specifically to international students who volunteered during specific crisis contexts such as the COVID-19 lockdown in Wuhan.

To address this gap, this study contributes to the literature by extending SDT to a unique group—international PhD students who were based in a foreign country during a public health emergency—and exploring how the three fundamental psychological needs (autonomy, competence, relatedness) shaped not only their intentions and motivation but also how these experiences foster resilience and social integration. According to the SDT perspective, these basic psychological needs play a critical role in how individuals engage with their community during challenging times like the COVID-19 pandemic (Alamer and Al Sultan, 2022).

In such crises, comprehending the motivations and resilience of international student volunteers becomes even more significant not only for academia but also for practical implications. Accordingly, the present study is guided by the following questions:

1) What motivated international students to volunteer during the Wuhan lockdown?
2) How did volunteering impact their resilience?
3) Did volunteering influence social integration among them? If so, how?

## 2 Literature review

### 2.1 Volunteering during pandemics and crises

Disasters, according to popular opinion, cause disturbance and disorder, with populations feeling helpless, terrified, or engaging in antisocial behavior such as looting. Individuals and communities, on the other hand, appear to become more linked than in “normal” times, commonly working to overcome disaster-related challenges (Scanlon et al., 2014).

Therefore, during an emergency or crisis, one of the most demanded resources is volunteers. During emergency situations, those who offer their time, expertise, abilities, and assets as volunteers will attain credibility and be integrated into the formal system by means of the volunteer recruitment initiatives implemented by agencies (Seah et al., 2021). In the recent pandemic, Mak and Fancourt (2022) have discerned three distinct categories of volunteering: formal volunteering, social action volunteering, and neighborhood volunteering. In the context of the COVID-19 pandemic, societal circumstances and personal beliefs affect people’s willingness to voluntarily follow COVID-19 preventative measures (Chan et al., 2021). According to Vignoles et al. (2021) and Stevenson et al. (2021), there is a positive correlation between community identification and volunteering amidst the COVID-19 pandemic. Furthermore, the research findings also indicated an indirect relationship between community identification and volunteers extending emotional support related to the pandemic.

### 2.2 International students and volunteering

Student volunteering is increasingly recognized as a key component of community engagement and experiential learning in higher education (Paull et al., 2015). This form of volunteerism benefits students, universities, nonprofit organizations, and society at large. Many educational institutions actively promote student volunteering, encouraging students to give back to their communities while pursuing personal goals (Haski-Leventhal et al., 2020). Research on student volunteering emphasizes its benefits: improved employability (Goodman and Tredway, 2016), resilience, personal skill development (Llenares et al., 2020; Williamson et al., 2018), external rewards such as opportunities to attend events or visit places for free, and getting certificates (Kifle Mekonen and Adarkwah, 2022). Students must also deal with time limitations, ambiguous motivations, confused identity, lack of supervision, and insufficient expectations (Lili and Yingjin, 2020; Cincalová and Cerná, 2021) as well as regret, dissatisfaction, and unmet expectations (Haski-Leventhal et al., 2020).

### 2.3 Understanding motivations for volunteering

Volunteer behavior involves individuals voluntarily responding to help-seekers after careful consideration (Snyder and Omoto, 2010). Key traits of voluntary service include being voluntary, free, public welfare-oriented, and altruistic (Shi et al., 2020). Although identifying different people who are volunteers is very helpful, it is equally important to know why people volunteer. Behavioral scientists’ and psychologists’ definitions of volunteer motivation vary substantially. Studies have identified multiple factors shaping volunteer motivations and willingness: Omoto and Snyder (1995) noted that motivations for volunteering range from altruism to self-interest. Clary et al. (1998) used a functional approach, suggesting that people may engage in similar activities even if their underlying motivations differ. Other motivations encompass the enhancement of social relations, acquiring of professional experience, alignment with organizational objectives (Mccormick and Donohue, 2019; Doidge and Sandri, 2019), skill development, and the cultivation of civic duty, altruism, and humanitarian principles (Schlesinger and Gubler, 2016; Hustinx et al., 2015).

### 2.4 Resilience and volunteering

Recent research in China highlights that the pandemic has led to increased symptoms of depression, anxiety, and stress (Chen and Huang, 2020; Lai et al., 2020; Wang et al., 2020), with higher rates of psychopathology and lower resilience in extreme stress compared to moderate events (Chen and Bonanno, 2020). Resilience refers to the ability of some individuals to withstand the problems they face (Köse et al., 2021). In other words, it is a pattern of competent behaviors demonstrated by people in situations of risk, which enables them to continue functioning well. Finally, resilience is multidimensional and complex, constructed through relationships, personal resources, meaning-making, leadership, culture, and knowledge (Blaney et al., 2021). Resilience is promoted by a number of factors, including personality as well as external factors such as social and interpersonal resources (Chen and Bonanno, 2020). Among volunteers, those who volunteer for more hours report higher resilience and volunteering may have a positive impact on volunteers’ well-being (Llenares et al., 2020). Also, a number of studies have shown that volunteering can provide many psychological benefits. According to Carlile et al. (2014), volunteerism is beneficial as a means of increasing psychological resilience and facilitating personal recovery among survivors of large-scale disasters, and providing emotional support to disaster victims allows volunteers to identify with those who are facing similar hardships.

### 2.5 Social integration through volunteering

Another concept that has been discussed in this research is social integration, which examines the degree to which individuals are engaged in different social relationships, such as being involved in social activities and interactions with communities. Social integration has been linked with a feeling of identification and belonging to one’s social life (Holt-Lunstad and Uchino, 2015). This concept generally refers to the engagement of people in social, cultural, economic, and political life in the community (Brydsten et al., 2019). Expanding this definition, different forms of social participation in the host society can affect an individual’s psychological cognition and identity (Xie et al., 2022).

According to prior research, international students enrolled in volunteer programs have higher levels of cultural adjustment and social interaction than those who are not volunteers (O’Neil et al., 2023). This is a sign that volunteering is a bridge for increased interaction that aids students not just for cultural adjustment but for their expansion of social networks. Unlike long-term immigrants seeking permanent residence, international students have temporary stays with specific academic goals, therefore social integration through short-term volunteer activities is particularly successful.

Social integration encompasses the feelings of belonging, inclusion, connectedness, participation, and recognition through volunteering, which are all crucial for international students’ adaptation to the new cultural and academic environment. Moreover, volunteer programs like community connection projects foster cross-national friendships. These experiences assist them to build social, cultural, and human capital, self-validation, and cohesion among varied groups (Manguvo et al., 2013; Gresham and Clayton, 2011). The establishment of these connections through volunteering enables students to gain practical insights into community dynamics, reinforcing their sense of belonging.

As Leonard and Onyx (2003) point out, by bridging diverse cultures, volunteerism is socially cohesive by creating a society based on a common cause. For international students seeking to adapt to the host society, this cohesion can have a positive impact on their adaptation and social integration process. Volunteering is a crucial means through which individuals can acquire experience of a variety of cultural aspects that may improve their effective integration and adaptation as well as generate positive feelings of inclusion, belonging, self-validation, and recognition.

### 2.6 Theoretical framework: self-determination theory

The framework of the Self-Determination Theory can provide an adequate context for establishing how the resilience toward voluntary behavior and social integration can be adjusted and utilized during times of difficulty, including a pandemic outbreak. SDT is the theory that individuals possess the capacity to make their own choices and navigate the consequences of these choices, which in turn plays a pivotal role in their overall well-being (Deci and Ryan, 2002). This theory posits that autonomy, competence, and relatedness are fundamental psychological needs essential for psychological growth and well-being (Deci and Ryan, 2000). Fulfilling these needs not only enhances an individual’s performance and voluntary engagement in activities but also strengthens their motivation (Ryan and Deci, 2017; Martela et al., 2021).

The relevance of SDT extends to how people respond to community challenges, such as those brought about by the COVID-19 pandemic, influencing their level of community involvement. Research indicates that applying SDT principles in interventions can elevate self-efficacy, foster personal responsibility, and diminish detrimental beliefs, thereby building resilience (Randall et al., 2022). Specifically, during times of crisis, SDT underscores the importance of meeting basic psychological needs like autonomy and competence for the development of resilience. Therefore, interventions grounded in SDT have been effective in enhancing resilience, reducing stress, and mitigating psychological distress among individuals during the pandemic. These interventions focus on autonomy, relatedness, and competence, helping individuals reconnect with others, boost self-confidence, and prioritize their psychological health, which in turn strengthens their resilience under adversity (Kulke et al., 2023).

Moreover, the application of the self-determination model by First Nations communities in Australia during the pandemic showcased the effectiveness of community-controlled responses that prioritize autonomy and self-governance, highlighting the role of SDT in promoting social cohesion (Anthony and McGrady, 2022). To strengthen social ties, promote integration, and implement pandemic-related actions, SDT—a theoretical framework that fulfills the criteria for autonomy, competence, and relatedness—is crucial.

In the context of international student volunteers in Wuhan, the application of SDT illuminates how volunteering can serve as a medium through which students find a sense of autonomy in their decision to contribute, competence through the skills and knowledge gained, and relatedness through connections with the community. These are the constituents of fostering resilience and will be part and parcel of gaining social integration, especially in gathering it during times of majorly sudden and unprecedented crises.

## 3 Materials and methods

The present study employed a qualitative method using semi-structured interviews including open-ended questions to collect data with the thematic analysis. Semi-structured interviews are used to acquire a detailed picture of a participant’s thoughts and perceptions. The researchers are able to “get deep and detailed insights into the lived experiences” through semi-structured interviews (Duff and Rankin, 2020). This approach was selected for accessibility, reliability, and flexibility (Braun and Clarke, 2006).

### 3.1 Sampling and data collection

The researchers conducted 14 semi-structured interviews with international students who were volunteers on the campus of Huazhong University of Science and Technology during Wuhan’s COVID-19 lockdown in order to identify factors that may influence their motivation for them to volunteer and their resilience and social integration during their voluntary activity. Due to the fact that some of the respondents had graduated at the time of data collection, some of these interviews were conducted via WeChat messaging, using a mix of voice messages and text, depending on participant preference. Data were collected between December 1, 2022 and February 15, 2023. Clarification was requested when cultural or language complexity arose. When participants used terms and idioms, that might be interpreted differently based on cultural background, the interviewer asked follow-up questions to clarify meaning and ensure accurate interpretation. This helped maintain the integrity of the data and supported the depth of participants’ narratives.

Snowball sampling is one of the most popular methods of sampling in qualitative research. In this sampling, the collection of samples begins with collecting data from one individual, and then that individual informs about the other, forming a chain that continues until there is a sufficient number of individuals to analyze (Bhardwaj, 2019). Sampling usually finishes once either a target sample size or saturation point has been reached (Parker et al., 2020). In this study, volunteers recommended other volunteers who were active at that time on the campus. Data saturation was reached when the final interviews generated no new insights. Numerous coding iterations were employed to identify saturation, with each iteration, the last three interviews confirmed rather than introduced new concepts. Table 1 provides a summary of the demographic characteristics of the participants. Despite the limited number of international student volunteers, the comprehensive nature of the interviews provided significant information.

**Table 1:** Demographics profile of research participants.

Indicator	*N*	%
Age		
≤30	2	14.30%
31–35	7	50.00%
3	3	21.40%
**>** ≤ 40	2	14.30%
Gender		
Male	7	50%
Female	7	50%
Volunteering experience prior to study		
Yes	10	71.40%
No	4	28.60%
Duration of residency in China		
Less than 1 year	2	14.30%
1–2 years	1	7.10%
3–4 years	8	57.10%
4–5 years	1	7.10%
More than 5 years	2	14.30%
Types of volunteer services provided		
Cleaning campus	3	21.40%
Leadership roles	2	14.30%
Ordering and disturbing food	5	35.70%
Medical checkups assistance	2	14.30%
Distributing protective masks	1	7.10%
Consulting and advising students	1	7.10%

Content validity of the interview questions assured its relevance and appropriate character. Based on a thorough examination of student volunteerism and Self-Determination Theory literature, the research team prepared the guide to assure interview question topic validity. The questions were designed to align with SDT’s core psychological needs: autonomy, competence, and relatedness.

Autonomy was explored through questions about participants’ willingness to volunteer (e.g., “What was your willingness to volunteer during the Wuhan lockdown?”), assessing their motivation. Competence was evaluated by questions about the benefits of volunteering (e.g., “How do you think the voluntary service helped you cope with that situation?”), showing participants’ perceived capabilities and development. Relatedness was examined through questions about social connection (e.g., “Did you feel more belonging to the community during voluntary service?”), focusing on emphasizing emotional intimacy and community support.

A pilot interview with one participant who met the inclusion criteria allowed researchers to evaluate question clarity and flow. A pilot was used to make minor changes to the interview guide before comprehensive data collecting. All interviews were conducted via WeChat; the student volunteer organizer and leader found first and then others suggested other volunteers. Each interview lasted approximately 30–40 min. Table 2 shows the interview questions.

**Table 2:** Interview questions guide.

No.	Questions
1	What was your willingness to volunteer during the Wuhan lockdown?
2	What advantages do you think your volunteer service had for you and others?
3	How do you think the voluntary service helped you cope with that situation
4	Did the voluntary service help you to be more optimistic about the situation? how?
5	Did you feel more belonging to the community during voluntary service? how?
6	Have you made new connections from volunteering at that time?
7	Did the voluntary activity help you experience integration or unity then? how?

In order to validate the findings from several perspectives, the experiences of the participants were cross-referenced with external sources such as reports, news articles, and studies on the lockdown’s impacts, ensuring the findings were corroborated through multiple sources.

### 3.2 Data analysis

The data were analyzed using thematic analysis, with NVivo 14 employed for coding. This flexible method identifies, analyzes, and reports patterns within qualitative data, allowing themes to emerge organically (Braun and Clarke, 2006). The analysis followed an inductive coding process, allowing themes to developed organically from the data without pre-established categories. The analysis followed a six-stage coding process:

#### 3.2.1 Phase 1: Familiarization with the data

The interview transcripts were read and reviewed several times line-by-line by the authors to get a general understanding of the text of the interviews and generate initial codes. This process involved breaking down the raw data into smaller, meaningful parts based on participants’ responses. The primary goal was to get key ideas and assign labels (codes).

#### 3.2.2 Phase 2: generating initial codes

During this phase, the raw interview data were coded manually with the help of NVivo which allows for a systematic approach to data analysis. Researchers reviewed each transcript, assigning initial codes based on the participants’ responses.

#### 3.2.3 Phase 3: searching for themes

The codes that were similar in terms of content and meaning were placed in a class and their relationship was determined. The aim of axial coding was to identify how different codes related to each other based on the context. For instance, codes like *“helping others”* and *“community welfare”* were grouped into the category *“altruism and community welfare,”* while *“managing stress”* and *“sense of purpose”* were categorized under *“coping mechanisms.”*

#### 3.2.4 Phase 4: reviewing themes

Themes were refined by cross-checking them with the coded extracts to ensure they were coherent, internally consistent. Codes like *“building connections”* and *“cultural awareness”* were categorized under *“social connectivity and cultural exchange.”*

#### 3.2.5 Phase 5: defining and naming themes

After phase 4, these concepts were grouped into three primary themes: *“motivation for volunteering,” “Resilience through volunteering,”* and *“social integration and community bonding.”* All steps were clarified during a team discussion, and in some cases, the names of the categories and subcategories was partially modified.

#### 3.2.6 Phase 6: producing the report

The final themes were written up with illustrative quotes to support the findings. To assess the reliability of the coding, an inter-rater agreement method was employed between two coders. Authors actively participated in coding the interviews, and the results of their coding were compared. Codes that were similar in both authors’ assessments were labeled as “agreement,” while those that were dissimilar were designated as “disagreement.” Subsequently, discrepancies were resolved through discussion, resulting in a 90% agreement rate.

### 3.3 Ethical considerations

This study was conducted in accordance with *the Declaration of Helsinki*. Ethical review and approval were not required in accordance with local legislation and institutional requirements of the College of Public Administration at Huazhong University of Science and Technology. Data were collected remotely and no physical interaction or intervention was involved. Participation was voluntary, with participants providing informed consent prior to involvement. Participants were communicated in advance about the research topic and objectives. They were assured of the confidentiality of their responses. To maintain focus and ensure relevant data collection, guidance was given during the interviews to ensure responses aligned with the research objectives, preventing tangential discussions.

## 4 Results

Three interrelated themes developed that reflected the experiences of student volunteers during the COVID-19 lockdown in Wuhan, based on Self-Determination Theory (SDT). Participants’ motivations show the interplay of autonomy (self-directed choice to volunteer), competence (skill development through volunteering), and relatedness (social connection). In turn, these motivations fostered resilience and shaped social integration, creating a cyclical reinforcement of psychological needs and outcomes. These themes, along with their sub-themes, are presented in Table 3.

**Table 3:** Data analysis.

Themes	Descriptions	Sub-Themes
Motivation for volunteering	Describe how volunteers were driven by altruistic motives, personal growth desires, and health-related needs.	- Altruism and community welfare - Personal and professional development - Personal health and the desire for physical activity
Resilience through volunteering	Explain how volunteering contributed to coping mechanisms and psychological resilience, emphasizing the role of community.	- Coping Mechanism - Psychological Impact of Community solidarity
Social Integration and Community Bonding	Discuss how volunteering facilitated connections between individuals from diverse backgrounds and fostered cultural exchanges.	- Social Connectivity - Cultural Exchange and Understanding

### 4.1 Theme 1: Motivation for volunteering

This theme explores motivations that led international students to engage in volunteer work during the Wuhan lockdown. These motivations include an altruistic drive to contribute to the community’s well-being (intrinsic), opportunities for personal and professional development, and maintaining physical health through activities (extrinsic). These motivations are consistent with the fundamental concepts of SDT.

*Altruism and Community Welfare (Relatedness):* Many volunteers were motivated by a desire to contribute positively to their community’s welfare, showing a commitment to helping others during a crisis. Moreover, based on the concept of SDT, *“relatedness”* emerged as identity-driven belonging which is deeply tied to participants’ self-concept as community members. For instance, P10 (female, 31–35) shared:

*“I like public work related to charity. I like to feel useful to society, especially in extremely difficult situations.”*

*"I just really wanted to contribute my part in whatever little way I could at the time to support all the systems and resources that were being put to make to keep everybody safe and help everybody stay comfortable."* As P4 (female, 31-35) explained.

These statements highlight how the participant’s sense of being *“useful to society”* and *“helping everybody stay comfortable”* reflected an intrinsic motivation to engage with and contribute to the well-being of their community, fulfilling the need for relatedness. The desire to contribute during crises emphasizes altruism as an internal drive, where participants derive personal satisfaction from helping others, even under difficult circumstances.

Similarly, P12 (male, 31–35) noted:

*“I wanted to give back to the community because I felt that in times of crisis, it’s important to step up and support each other. It made me feel like I was making a difference, even if it was in a small way.”*

Here, the act of “*giving back*” reflects a sense of relatedness and belonging as well as contributing to collective well-being. Moreover, the desire to *“step up and support each other”* reflects not only personal commitment but also recognition of the social contract that binds communities together during crises. This reflects how the participant’s feeling of being *“useful to society”* aligns with their values in the lockdown context. Contributing during crises reflects altruism as an inner drive, through which participants derive personal satisfaction from helping others, even under adverse conditions.

*Personal and Professional Development (Competence):* Many students viewed volunteering as an opportunity for personal and professional growth, helping them develop practical skills like stress management, communication, and teamwork. Additionally, *“Competence,”* one of the components of SDT, was expressed through self-efficacy, as the participant acquired new skills and effectively navigated challenging environments. As P9 (male, ≤30) noted:

*"It further helped me to enhance my management skills in emergency situations and develop communication/negotiation skills to counsel the mentally disturbed."*

Similarly, P3 (male, ≤30) explained:

*“Another benefit for me to get to see how my little contribution can make a difference. My personal development such as self-confidence and self-esteem flourished.”*

Here, the participant clearly connects volunteering with internal competence building — confidence and esteem — grounded in values and contribution.

P10 (female, 31–35) also reflected on how volunteering strengthened her personal sense of purpose and inner confidence:

*"I got more courage and heroic feeling; I think it's best feeling when you feel that doing something important for society."*

These statements demonstrate how volunteering not only enhanced professional skills during the crisis, but also achieved personal growth and strengthened their competencies, including confidence, and a sense of impactful service. Such skill development directly addressed the need for competence.

*Personal Health and the Desire for Physical Activity (Autonomy):* With the physical mobility controls of the lockdown, volunteering offered a platform for physical activity to aid in the sustenance of mental and physical health. Engaging in volunteer activities to maintain physical health during lockdowns demonstrates the importance of “*autonomy”* in SDT as the participants emphasize it through self-directed choice. For example, P1 (female, >40) stated in response to a question regarding her motivation:

“To exercise my body after long stays indoors. To keep the environment clean, healthy, and safe for students.”

P5 (female, 31–35) noted: *"Coming out to keep the entire campus environment clean afforded us the opportunity to exercise our bodies, move around, and get some fresh air."*

This autonomy in choosing roles supported mental and physical health, a key SDT driver. The possibility to *“move around”* and *“get some fresh air”* shows how students looked for means to remain active while contributing to their community.

P11 (female, 31–35) described a similar experience using a meaningful metaphor:

*"The room [of dormitory] was like a cage and I could get a chance to come outside of this cage [through volunteering] for some time every day."*

The expression of *“the room as a cage”* reinforces how volunteering served as a self-directed way to physically escape, providing physical movement and autonomy during lockdown.

A report from the Royal Society (2020) noted that restrictions on public transportation and movement were so rigorous that only necessary trips were allowed, leading residents to seek alternative ways to stay active. For many participants, simple tasks like cleaning provided not only physical exercise but also a mental health boost, reducing the sense of confinement. Research indicates that physical activity levels dropped drastically during the pandemic (Ding et al., 2021) and that many residents participated in home-based or community events to preserve their health (Zhou et al., 2021; Qin et al., 2020), hence these results imply that international students turned to volunteering as another way of staying active.

### 4.2 Theme 2: Resilience through volunteering

Motivation driven by SDT needs helped participants develop resilience through volunteering, emphasizing both individual and collective aspects of psychological adaptation during the lockdown. This nuance is explored in two sub-themes: *Coping Mechanisms*, which describes internal, self-regulatory strategies used to manage stress and maintain mental health, and *Psychological Impact of Community Solidarity*, which describes the emotional and relational benefits of shared experiences and mutual support in the volunteering community.

*Coping Mechanism*: This sub-theme mainly focuses on individual strategies that kept participants engaged and distracted from the stress and anxiety caused by lockdown. Reports from Wuhan highlighted how movement and social restrictions led to widespread psychological strain (Royal Society, 2020). Community service became a key coping mechanism, aiding in stress, anxiety, and depression management through collective action and a sense of usefulness (Royal Society, 2020; Li M. et al., 2023).

P14 (female, 36–40) described volunteering as a lifeline:

“*Volunteering during the lockdown was like a lifeline. It gave me a sense of purpose and kept my mind occupied. It helped me deal with the stress and isolation because I knew I was contributing to something bigger than myself.*”

The metaphor *“lifeline”* emphasizes that volunteering was essential for emotional and psychological survival during lockdown, serving not only as an activity but also as a coping mechanism. It provided guidance and structure, reducing the adverse effects of isolation. Regional reports have observed similar patterns of resilience due to intentional actions during the outbreak (Wong et al., 2015).

*“With the lockdown … within two-weeks I started feeling my brain stopped working … After one month… I started to feel anxiety and depression… Then I asked my hostel administration to add my name in the list of volunteers. My depression level came down a bit… I was used to be very busy before that situation.”- P 11 (female,* 31–35*).*

This participant stated how losing her routine during lockdown caused mental exhaustion, unhappiness, and anxiety. Volunteering helped her feel better by bringing back structure, social connection, and a sense of individual purpose.

P1 (female, 31–35) expressed the emotional load and the relief derived from volunteering:

“*This enabled me fight depression and loneliness.*”

These statements demonstrate how volunteering not only served as a daily task, but also as a critical emotional outlet for students confronting isolation, anxiety, and mental fatigue.

*Psychological Impact of Community Solidarity:* This sub-theme shows the collective aspects of resilience that emerged from working with others during the lockdown. It demonstrated significant benefit in fostering emotional resilience through social solidarity. It can also reflect relatedness aspects of SDT since shared experiences and mutual support contributed to the development of a stronger sense of community.

As P12 (male, 31–35) shared:

"*We helped each other and saw every other day come and pass… By helping each other and serving our brothers and sisters, we are able to see the bright day; I think this is what coping up means to me.*"

The metaphor of a *“bright day”* brings into view how volunteering and community support fostered such collective resilience. Emotional strength from these shared experiences is well-documented in crisis situations. Reports from Wuhan show that mutual aid, including sharing resources on platforms like WeChat, helped strengthen emotional resilience during the lockdown (You et al., 2023).

Additionally, P7 (male, >40) stated:

"*It creates a supportive mental and emotional atmosphere for international students.*"

P5 (female, 31–35) also emphasized:

“*Those of us who were volunteering had the opportunity to at least interact even though it’s from a distance… we were just happy to do it, to see each other doing the work together. We were encouraging each other.*”

This statement reflects the emotional support gained from shared experiences during the lockdown, which helped fulfill the need for relatedness. Volunteering within communities helps to strengthen resilience by providing social support and a shared sense of purpose (Southwick et al., 2014).

### 4.3 Theme 3: Social integration and community bonding

Volunteering fostered social outcomes that fed back into SDT needs. This theme reflects how volunteering during the lockdown promoted social integration and community bonding among international students from diverse backgrounds.

*Social Connectivity:* Despite all the difficulties brought up by physical distance, the volunteers built new social contacts enhanced from this period. As these participants mentioned:

*"I met new people when I volunteered. We continue to have a strong relationship with a few of them till today." -* P8 (female, 31-35)

*“I made some new friends and experienced a friendly environment. We started discussing the issues and encouraging each other. We were united.”*- P11 (female, 30–35).

*P6* (male 36–40) stated:

*“Besides simple greetings, I connected with some students, and we are still attached.”*

These statements show how common experiences during volunteering bridged physical distance and helped participants to create close relationships, fulfilling the need for relatedness by making new social connections and facing a friendly community. Moreover, the term *“strong relationship”* reflects the core of volunteering and emphasizes how group activity during crisis can break down social boundaries and advance closer relationships. Volunteering during disasters can assist to remove social barriers, leading to strengthen ties in the community (Kneale et al., 2023).

*Cultural Exchange and Understanding:* Volunteering also promoted understanding of culture as students from different cultures could learn from one another. This promoted a greater feeling of relatedness by means of shared experiences and by eliminating boundaries between cultures. As P5(female, 31–35) noted:

*“I made new connections and learned about the mindsets of students from different backgrounds during the environmental disruption."*

Similarly, P12 (male, 30–35) mentioned:

*“The more we close to society, the more we are attached to the people. It gave me a feeling of an international family”.*

P2 (male 36–40) shared a similar perspective:

*“Most of the other volunteers were not from my college or country… it was a good experience and it helped us integrate better.”*

P3 (male, ≤30) added:

*“We are international students from different countries with different cultures. We have to help each other to survive."*

Or P8 (female, 31–35) stated:

*"It taught me there is no better solution to a problem but working together. The lesson of unity not only came from the university, but from the community, society, state, and country. Volunteers, local communities, hospitals, and authorities worked together for Wuhan. Many doctors from different provinces came to help. I experienced this togetherness closely as a volunteer. There can’t be any better life lesson of unity than this."*

These quotations indicate how volunteering helps to promote cultural interaction and mutual understanding, therefore allowing volunteering to link across boundaries of culture and ultimately lead to contribute to a sense of relatedness. This common experience improved social integration and fostered community (Khvorostianov and Remennick, 2017). Such outcomes reflect broader trends showing how volunteering and community efforts during crises promote inclusivity and social cohesion (Institut National de Santé Publique du Québec, 2021).

P13 (male, 35–40) summarized this theme well:

*“Volunteering helped me integrate into the international student community. I was able to learn about different cultures,… enriched my experience during the lockdown.”*

Three themes emerged from international students’ volunteering experiences during Wuhan’s COVID-19 lockdown, structured around Self-Determination Theory (SDT). First, motivation was driven by fulfilling SDT needs: *autonomy* (self-directed roles for physical health), *competence* (skill development), and *relatedness* (altruistic community contributions). Second, sustained motivation fostered resilience, enabling coping through structured tasks and community solidarity, which reinforced participants’ sense of purpose and efficacy. Additionally, volunteering promoted social integration, breaking cultural barriers and fostering enduring bonds, deepening relatedness through shared experiences. These outcomes cyclically replenished autonomy, competence, and relatedness, creating a self-reinforcing model where psychological needs and collective well-being mutually sustained engagement.

## 5 Discussion

This study addressed how international student volunteers supported resilience and social integration during Wuhan’s COVID-19 lockdown and investigated their motivations through their engagement. The findings identified key motivation factors, including altruism, personal growth and development as well as health-related needs, which align with Self-Determination Theory (SDT). This theory states that individuals become most motivated when their psychological needs for autonomy, competence, and relatedness are met (Deci and Ryan, 2000).

**Figure 1:**
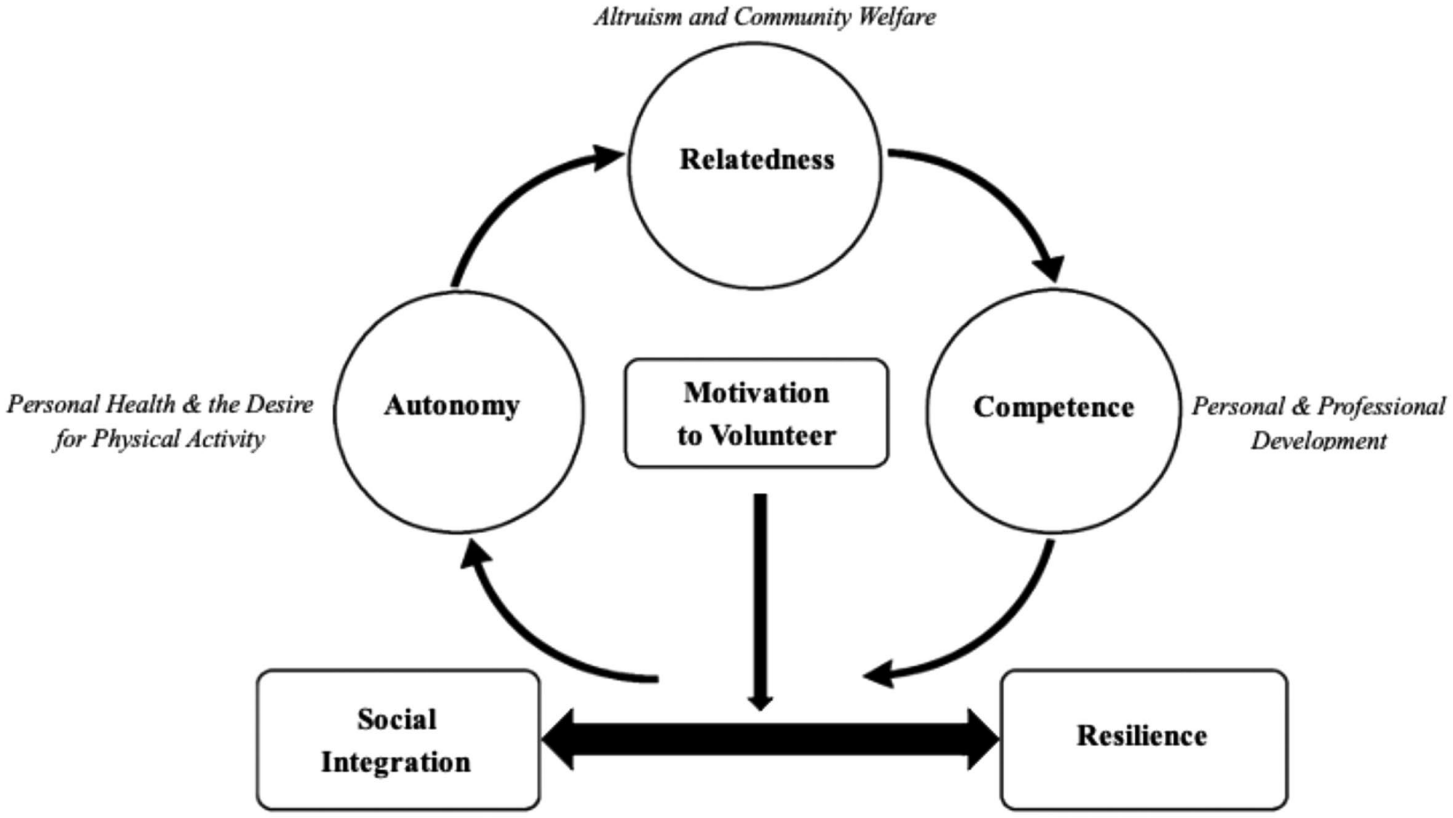
SDT model of volunteer motivation, resilience, and social integration.

These fundamental psychological needs do not function independently. Their interaction plays a vital role in sustaining motivation and fostering resilience and social integration during crises. In this study, when participants engaging in volunteer activities and experienced autonomy, competence, and relatedness, the satisfaction of each need appeared to mutually reinforce the others. This interplay creates a self-reinforcing cycle of intrinsic motivation: satisfying one need increases the satisfaction of the others, hence generating a strong internal drive to continue volunteering even under difficult circumstances. In turn, motivated engagement led to outcomes like social integration and resilience, which helped to confirm the volunteer commitment. Figure 1 shows the dynamic model developed from this study showing how the interaction among different psychological needs drives motivation and supports empowerment and development in crisis situations.

The findings emphasize that students’ need to help others and emotionally connect to others was the main factor influencing their decision to volunteer. Specifically, within the sub-theme of altruism and community welfare, the results are consistent with SDT’s concept of relatedness, which explains how individuals seek meaningful social connections via their actions. Prior studies confirm that relatedness is a major motivator of volunteer activity, particularly in crisis situations where community solidarity becomes a strong incentive (Schwarz et al., 2022; Li F. et al., 2023). For international students, who were at risk of social isolation during lockdown, volunteering served as a channel for emotional support, and group membership—reinforcing motivation and fostering social integration.

Students also developed competence, gaining practical skills and confidence through meaningful contributions during a crisis. Many participants reported acquiring new practical and emotional skills, including stress management, teamwork, and leadership. This reveals how the personal and professional lives of volunteers are impacted by their self-determination (Solansky, 2015). Volunteering likely increased the participants’ abilities to manage stress, communication, and skills in teamwork. This is consistent with earlier research showing that volunteering during the 2015 European refugee crisis is quite different from volunteering under normal circumstances. In these environments, volunteers frequently exhibited considerable self-organization, demanding the development and enhancement of practical management and coordination skills for effectiveness (Simsa et al., 2018). These skills are especially advantageous in high-pressure settings, where decision-making and adaptability are essential. Whittaker et al. (2015) also emphasize that these experiences cultivate adaptability, thereby enhancing volunteers’ preparedness for professional challenges. Autonomy and competence function first as short-term coping tools—reducing immediate stress—and over repeated experiences build up self-efficacy and problem-solving skills that underpin lasting resilience.

Moreover, participants indicated that engaging in physical exercise through volunteer work contributed to their mental and physical well-being. Participating in these activities, especially during lockdowns, demonstrates the importance of autonomy in self-determination theory. Individuals who stay physically active during stressful times can reduce anxiety and stress (Stukas et al., 2016; Pettigrew et al., 2015). Many participants highlighted that when other freedoms were restricted, volunteering provided them agency by means of “move around,” “stay busy,” and “escape the cage” of dormitory isolation. These expressions illustrate that autonomy is not just about independence, but having control over one’s actions even in constrained circumstances (Precht et al., 2023; Anicich et al., 2020). Research also indicates that when volunteers view their activities as autonomous and meaningful, their intrinsic motivation increases (Haivas et al., 2012; Lorente-Ayala et al., 2020). Research also indicates that autonomy healthy lifestyle compliance, especially during pandemics (Williams et al., 2011; Anicich et al., 2020). This suggests that volunteering improved participants’ immediate well-being and promoted long-term healthy habits. Organizations should acknowledge volunteering’s health benefits and build physical and mental resilience, as Volunteer movement and involvement boosts nonprofit productivity, motivation, and retention. It appears that while many volunteers are driven by inner satisfaction and altruism, organizations should also recognize and encourage extrinsic motives to keep them volunteering. Furthermore, the study shows that helping during disasters is a transformative learning experience that gives participants valuable skills for personal and professional development. This highlights the need for nonprofits to support volunteer skill development to make crisis volunteering both humanitarian and developmental.

It is through this pattern that a sustained reinforcing cycle emerges. Emotional safety that was achieved through relatedness allowed the students to internalize competence through service. This developing sense of competence made them more self-assured in guiding themselves in what to do, achieving autonomy. Autonomy in turn fueled commitment, enhanced membership in the community, and promoted ongoing advancement—ending the cycle. When a need was fulfilled, it served to enable the next to be fulfilled, building a self-reinforcing cycle of engagement. This corresponds to the principle of self-determination theory that the needs in fact are interdependent and synergistic in nature (Ryan and Deci, 2017).

Together, these fulfilled needs fostered resilience, enabling students to adapt psychologically, manage stress, and maintain well-being despite adversity (Liu and Huang, 2021). These are consistent with research indicating that community service can reduce stress and boost psychological resilience (Lenares et al., 2020; Wong et al., 2015; Southwick et al., 2014). Volunteering after major disasters enhances psychological resilience, aiding in personal recovery (Carlile et al., 2014). SDT describes the capacity to maintain or restore mental health in the face of adversity. Research also showed that satisfaction of the core psychological needs of autonomy, competence, and relatedness improved volunteering and national responsibility (Martela et al., 2021). For instance, this aspect was reflected in the engagement of Saudi students during the pandemic whereby the key role in determining volunteer behavior is the fulfillment of fundamental psychological needs for autonomy, competence, and relatedness (Alamer and Al Sultan, 2022). This study found that international student volunteers in Wuhan were more resilient when they felt autonomous, learned new skills, and made supportive contacts. This finding reinforces the idea that the three needs jointly mediate the link between crisis volunteering and resilience. This implies that non-profit organizations include structured volunteer work in their crisis response efforts so that volunteers not only prove useful but also gain psychological support from their work.

Finally, the study shows that volunteers’ social integration and community cohesion helped them overcome social isolation, which was crucial for international students stocked during the lockdown. This supports Self-Determination Theory (SDT), which states that well-being depends on feeling connected to others (the need for relatedness) (Geng et al., 2022). Volunteering brought students from different cultures together with a same goal, creating a deep sense of connection. Even during a pandemic, volunteerism connected people and formed lasting bonds. Cultural interaction reduced loneliness and helped students integrate into the host community. A volunteer framed it as being part of “an international family,” saying that the more they participated in society, “the more we are attached to the people.” This result was in line with earlier findings that volunteering may enable social integration for new or vulnerable individuals or groupings of people (O’Neil et al., 2023). Similarly, Ruiz and Ravitch (2023) found that formal volunteering helps first-generation immigrants in the U. S. overcome social isolation. Volunteering increases social integration and generates support groups for immigrants and minority students even in non-pandemic environments (e.g., Khvorostianov and Remennick, 2017; Manguvo et al., 2013).

The interaction between relatedness, competence, and autonomy provides a complete model to explain how international student volunteers in Wuhan responded to crisis situations. Meeting these three needs provided a mutually reinforcing motivational process that not only resulted in ongoing participation but also in resilience and social integration. This research extends the application of SDT by demonstrating how its foundational constructs work in conjunction in the face of crises, and highlights the double value of volunteering as a humanitarian contribution and as a psychologically fulfilling experience.

### 5.1 Contribution and directions for further research

The purpose of this study is to understand the motives of international student volunteers whose voluntary efforts contributed to the society in boosting resilience and integration during the lockdown phase of COVID-19 in Wuhan and reflect self-determination theory (SDT). It may contribute to a better understanding of volunteering in circumstances of crisis and provide insightful suggestions on how to manage and create volunteer programs that will be even more successful in the future.

The findings of this research have important implications for both academics and practitioners. First, it informs an understanding of what motivates international students to volunteer in emergency settings such as the COVID-19 pandemic and that is a combination of both altruistic and self-serving motives. Understanding motives of student volunteers could have strategies developed to promote resilience. Institutions may encourage this by helping them set an environment where students may come first for altruistic purposes, and second for personal growth, and physical activity.

Secondly, through employing the self-determination theory (SDT), individuals may comprehend how satisfying the psychological requirements of autonomy competence, and relatedness might perhaps enhance resilience and promote social integration among volunteers. This research addresses the gaps in Self-Determination Theory and demonstrates how integrating its concepts into educational practices may enhance the academic and personal growth of international students, resulting in a more supportive and empowered learning environment.

Thirdly, the research has even identified the remarkable importance of volunteering in the process of building resilience and sustaining social integration and belongingness by international students in crises. This highlighted that involvement in volunteer activities might be associated with gaining long-term psychological and social benefits. Therefore, this information will be highly useful to policymakers and scholars seeking to understand how to implement volunteering interventions, which can assist in increasing mental well-being and community connectedness during disasters.

### 5.2 Limitations and future research

This research has some limitations. First, the small size of the sample may restrict the generalizability of the findings. This is mainly owing to the limited number of volunteers during that the lockdown and due to the fact that it relied on those who consented to participate. Consequently, the sample may not represent the full range of experiences and motivations of all international students who volunteered.

Second, Interviews took place from December 2022 to February 2023, subsequent to the initial Wuhan lockdown early on 2020. The time lag could have introduced memory recall bias, which could have compromised the richness and accuracy of memory of the participants. The delay was largely necessitated by strict international travel measures and prolonged campus shutdowns. Also, most research work was called off during the pandemic. At that moment, the initiation of data collection was impossible due to a mix of emotional pressure and logistic constraints brought by the crisis.

Third, studying voluntary behavior and its outcomes in a particular context might not imply that it can be generalized to other settings. Given the specific context of this study -Wuhan during the COVID-19 lockdown-, the findings might not completely apply to other demographics or settings. Further research is necessary to provide the evidence supporting this study’s results. For example, comparative studies could show how different cultural or crisis-related contexts shape volunteer motivations and outcomes.

Lastly, Interviews were conducted via WeChat, due to COVID-19 restrictions and the international dispersion of participants. Although this approach may have affected the depth and tone of replies, this method allowed for flexible and accessible communication. Future studies might combine online and in-person approaches to gather complex narratives.

## 6 Conclusion

This study shows how international student volunteers in Wuhan built resilience and promoted social integration during the COVID-19 lockdown through volunteer work. By applying Self-Determination Theory (SDT), the research reveals how the fulfillment of the three psychological needs—autonomy, competence, and relatedness—not only motivated students to volunteer but also supported their psychological adaptation and social bonding in a crisis setting. These findings support the theoretical basis of Self-Determination Theory (SDT) by expanding its applicability to community-based crisis volunteerism experiences. Furthermore, by recognizing SDT for understanding pro-social behavior and resilience development in uncertain and emergencies circumstances, this study adds to the body of knowledge as well as offers an effective way for enhancing support and participation in the context of international students during next crises.

## Data Availability

The raw data supporting the conclusions of this article will be made available by the authors, without undue reservation.
